# Non-pharmacological Interventions for the Prevention and Progression of Diabetic Foot Ulcers in Individuals With Type 2 Diabetes Mellitus: A Scoping Review

**DOI:** 10.7759/cureus.100710

**Published:** 2026-01-03

**Authors:** Megumi Kamogawa, Kohei Kajiwara, Hideaki Sakuramoto, Koji Yamamoto

**Affiliations:** 1 Faculty of Nursing, Japanese Red Cross Kyushu International College of Nursing, Munakata, JPN; 2 Faculty of Nursing, Shimonoseki City University, Shimonoseki, JPN; 3 Division of Faculty Development, Nursing, Kindai University, Osakasayama, JPN; 4 College of Nursing, Japanese Red Cross Kyushu International College of Nursing, Munakata, JPN

**Keywords:** dfu, non-pharmacological intervention, prevention, recurrence, scoping review

## Abstract

Diabetic foot ulcers (DFUs) are a common complication of diabetes, significantly impairing patients' quality of life (QoL) and imposing a heavy economic burden. Previous reviews on the prevention of DFUs and their recurrence have indicated that educational interventions alone cannot definitively reduce the incidence of new DFUs or lower limb amputation rates and that non-pharmacological therapies using medical devices face challenges such as cost, the need for technical training, and infrastructure development.

This study systematically mapped the existing literature on non-pharmacological therapies, excluding medical devices and medications. Specifically, interventions involving machinery were excluded. The aim was to identify strategies that prevent delayed progression and recurrence of diabetic foot ulcers (DFU) in patients with type 2 diabetes.

The review focused on interventions with high feasibility, cost-effectiveness, and sustainability, such as patient education, behavior modification, monitoring, and multidisciplinary collaboration. These approaches are expected to contribute to the development of comprehensive and practical strategies that address current knowledge gaps, improve patient outcomes, reduce healthcare costs, and enhance the quality of life for individuals with diabetes.

An exploratory review was conducted using a combination of controlled vocabulary and free text terms related to "type 2 diabetes," "ulcer," "intervention," and "prevention." Data were collected from PubMed, the Cochrane Central Register of Controlled Trials, and Ichushi-Web.

Eleven studies were evaluated. The intervention methods used were face-to-face methods by healthcare professionals and methods utilizing information and communication technology (ICT). The outcome measures of the interventions varied, including changes in patients' cognitive behavior, assessment of quality of life, examination of the condition of the lower limbs, such as ulcer healing and skin condition of the feet, and evaluation of diabetes management. This review emphasizes that non-pharmacological therapies, particularly those incorporating multidisciplinary educational interventions involving nurses, may be effective in preventing diabetic foot ulcers (DFUs) and preventing their recurrence. Future research should aim to establish standardized outcome measures and evidence-based guidelines for integrating face-to-face and ICT-based multidisciplinary educational interventions.

## Introduction and background

The worldwide incidence of diabetes continues to rise, reaching 537 million. The prevalence of diabetic foot ulcer (DFU) is estimated to be 6.3%, with a reported rate of 5.5% in Asia [1]. Diabetic foot ulcers (DFUs) are one of the most serious complications of diabetes. In terms of pathophysiology, the prevalence of DFU is higher and more likely to worsen in type 2 diabetes [1,2]. When blood sugar control is inadequate, ulcers can develop due to neuropathy, ischemia, and minor trauma [3]. Patients experience a decline in quality of life (QoL) and may encounter lower limb infections or amputations [4]. The economic burden is substantial, with significant medical expenses rendering DFU prevention a major challenge [5]. Furthermore, the recurrence rate remains high, with approximately 40% within one year and over 60% within three years [6], underscoring the urgency of prevention, early intervention, and strategies to delay progression.

Previous reviews have yielded several insights across primary, secondary, and tertiary prevention. In primary prevention, identifying risk factors, patient education, and promoting self-management of foot care are emphasized [7,8]. In secondary prevention and delaying progression, multidisciplinary team management involving collaboration among healthcare professionals has been shown to be effective [9]. In tertiary prevention, continuous patient education and monitoring are emphasized [10]. However, three critical gaps remain: first, there is no definitive evidence that educational interventions alone reduce the incidence of new DFUs or lower limb amputation rates [10]. Second, non-pharmacological therapies utilizing medical devices (such as negative pressure wound therapy, hyperbaric oxygen therapy, and electrical stimulation therapy) have demonstrated efficacy, but their high costs, technical training requirements, and infrastructure requirements limit their feasibility and sustainability in many clinical settings [11,12]. Third, heterogeneity in outcome measures across studies hinders the standardization of evidence [10].

Against this backdrop, this study aims to systematically map the literature on non-pharmacological therapies that do not use medical devices or pharmacological agents, targeting the prevention, delay of progression, and prevention of recurrence of DFU in patients with type 2 diabetes. By excluding device-based and medication-based strategies, this scoping review focuses on potentially more feasible, cost-effective, and sustainable approaches such as education, behavior modification, monitoring, and multidisciplinary collaboration. This review is expected to clarify existing knowledge gaps, identify feasible intervention strategies, and contribute to improved patient outcomes, reduced healthcare costs, and enhanced quality of life for patients with diabetes.

## Review

Materials and methods

Study Design and Protocol

This scoping review was conducted following the methodological framework proposed by Arksey and O'Malley [13], and further refined using the Joanna Briggs Institute (JBI) Manual for Evidence Synthesis [14]. The reporting of this scoping review adheres to the Preferred Reporting Items for Systematic Reviews and Meta-Analyses extension for Scoping Reviews (PRISMA-ScR) guidelines (Supplementary Table 1) [15]. A protocol was not registered for this review, as registration is not mandatory for scoping reviews that aim to map available evidence rather than evaluate specific interventions [15].

Research Question

This scoping review addresses the following questions. Is it possible to identify comprehensive non-pharmacological interventions applicable across care, including preventive strategies before DFU onset and prevention and control of progression after onset? What is the scope and nature of non-pharmacological interventions adopted in both preventive strategies and treatment approaches for DFUs?

Population-Concept-Context (PCC) Framework

The following PCC framework guided our review as prescribed by the JBI scoping review methodology [14]: Population (P) consisted of patients with type 2 diabetes mellitus, Concept (C) comprised non-pharmacological interventions for DFU in preventive strategies and therapeutic approaches, and Context (C) included any healthcare setting, encompassing both acute and chronic care environments.

Search Strategy

A comprehensive literature search was conducted across three electronic databases: the Igaku Chuo Zasshi database (Ichushi-Web), MEDLINE via PubMed, and the Cochrane Central Register of Controlled Trials. Based on our established PCC framework, we developed search strategies using a combination of controlled vocabulary (MeSH terms) and free text terms related to "diabetes mellitus," "ulcer," and "intervention," or "prevention." For example, the PubMed search strategy included the following: ("Diabetes Mellitus, Type 2"[Mesh] OR "type 2 diabetes"[tiab]) AND ("Diabetic Foot"[Mesh] OR "foot ulcer"[tiab] OR "diabetic foot ulcer"[tiab]) AND ("Prevention and Control"[Mesh] OR "prevention"[tiab] OR "intervention"[tiab] OR "education"[tiab]). Full search terms are presented in Supplementary Table 2. To maximize coverage of relevant literature, no restrictions were applied regarding publication date or language. Studies involving surgical procedures, the utilization of medical devices, or the application of wound dressings as interventions were excluded. These searches were conducted in December 2024.

Eligibility Criteria

We developed definite inclusion criteria in accordance with the research question and PCC framework to guide the selection of relevant studies. The studies included patients with type 2 diabetes who developed DFU and received non-pharmacological therapy. Furthermore, among non-pharmacological therapies, we excluded studies involving surgical interventions, negative pressure wound therapy, electrical stimulation, hyperbaric oxygen therapy, and offloading devices, focusing instead on reviews that addressed education and care by healthcare professionals. The study designs included in the review were randomized controlled trials (RCTs), non-RCTs, crossover trials, single-group before-and-after studies, and cohort studies. All publications were included, regardless of language. The following study designs were excluded: review articles (systematic reviews and meta-analyses), letters, expert opinions, research protocols, conference proceedings or abstracts, cross-sectional studies, case series, case studies, books, and qualitative studies.

Study Selection Process

Citations were uploaded to Rayyan (http://rayyan.qcri.org), and duplicates were removed. Two pairs of reviewers (MK and KY, and KK and HS) performed the screening process in two stages, in accordance with the JBI guidelines [14]. First, titles and abstracts were screened independently by two reviewers against the inclusion and exclusion criteria in alignment with our PCC framework. When eligibility could not be determined based on title and abstract screening, full-text articles were obtained for further assessment. For secondary screening, the full text was uploaded to Rayyan and independently evaluated by two reviewers based on the inclusion and exclusion criteria. Any disagreements between reviewers were resolved through discussion to arrive at a consensus. When consensus could not be reached through discussion, a third reviewer was consulted to make the final decision based on the predefined eligibility criteria.

Data Extraction

A standardized data extraction form was developed based on the objectives of the review. For each included study, we extracted study characteristics (authors, year of publication, country, and study design), population characteristics (age, sex, diabetes duration, and comorbidities), details of non-pharmacological interventions (type, duration, frequency, and provider), outcomes related to DFU prevention or treatment, findings related to healthcare professionals' awareness of DFU risk, and identified barriers to risk recognition and intervention implementation. Data was retrieved by one reviewer from a study included in our scoping review, and discrepancies, if any, were resolved through discussions. In the absence of a consensus, a second reviewer proceeded with the assessment.

Data Synthesis

Given the exploratory nature of this scoping review, we employed a narrative synthesis approach as described by Popay et al. [16]. The extracted data were organized thematically according to the research questions. The findings were summarized descriptively, highlighting patterns, gaps, and similarities across the studies. Tables and figures were used to present the study characteristics and main findings in a structured format, in line with recommendations by Arksey and O'Malley [13].

Ethics and Dissemination

Ethical approval was not required for this scoping review as it involved analysis of previously published studies. The findings will be disseminated through publication in a peer-reviewed journal and presentation at relevant conferences to inform clinical practice and future research, as recommended by the PRISMA-ScR guidelines [15].

Results

Study Selection and Characteristics

**Figure 1:**
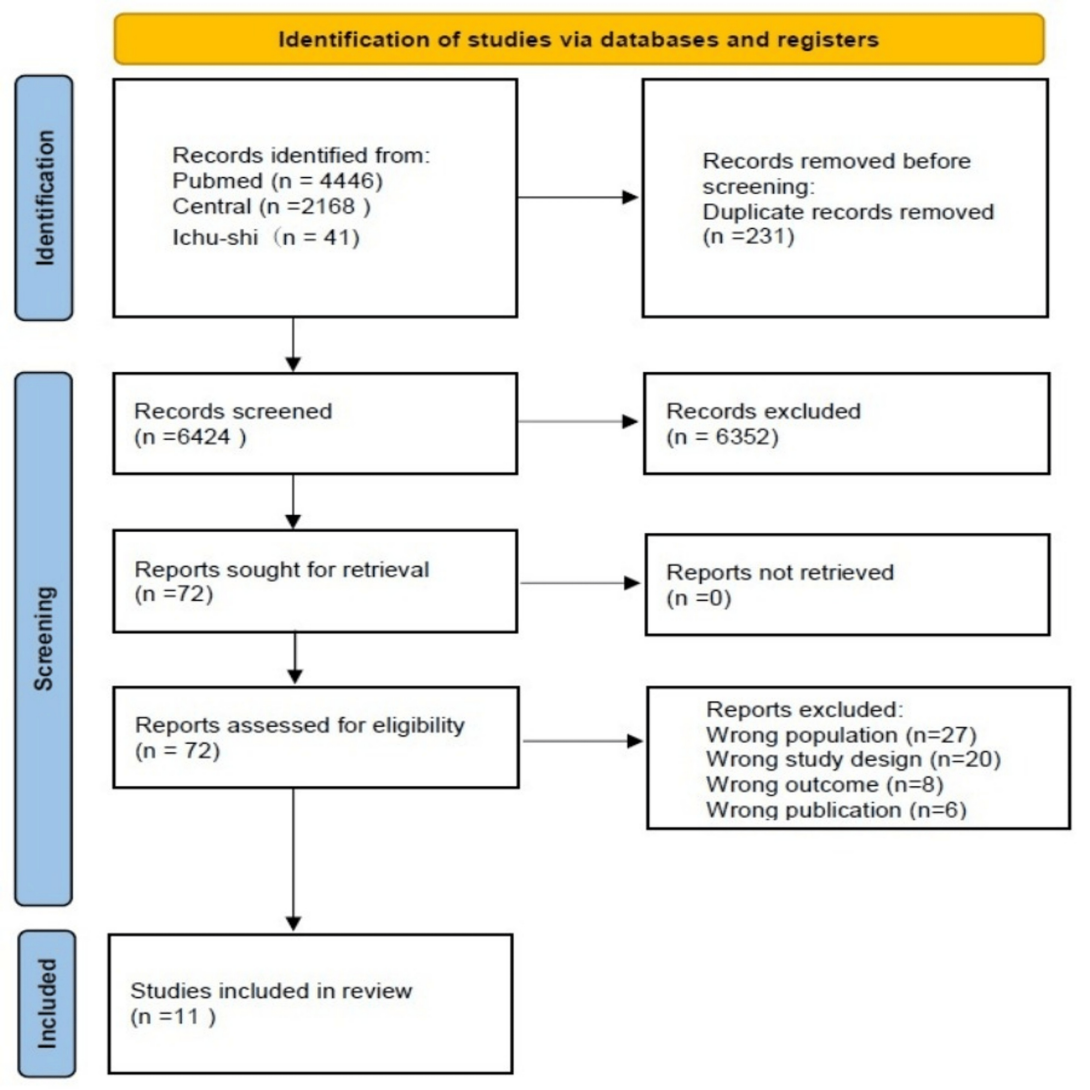
Flowchart of the study selection process

Figure 1 shows the literature search strategy and outcomes. Following the exclusion of duplicate records, a total of 6,515 studies were screened based on titles and abstracts, yielding 73 eligible studies. After full-text review, 62 studies were excluded, resulting in 11 studies that met the inclusion criteria for this review. These studies were conducted between 1993 and 2022.

Table 1 presents the characteristics, design, and source of origin of the studies. The studies were conducted in Asia (n = 4) [17-20], the Middle East (n = 3) [21-23], North America (n = 3) [24-26], and Italy (n = 1) [27]. The study designs were distributed as follows: quasi-experimental methodologies (n = 4) [17,19,22,23], RCTs (n = 5) [18,20,25,26], and a non-randomized controlled trial (n = 2) [24,27]. The study population exhibited considerable diversity in terms of residential settings, treatment environments, and risk levels for developing DFUs, as determined by the specific aims and eligibility criteria of each study.

**Table 1:** Characteristics of the studies included in our scoping review

Author and year	Country	Study design	Number of patients (intervention group/control group)/participants
Nguyen et al. (2019) [17]	Vietnam	Controlled, pre-test/post-test quasi-experimental design	119 (59/60)/Participants must have at least two months of treatment and follow-up at the adult clinic, be proficient in Vietnamese, and reachable through telephone. They should have a low risk of foot ulcers and will be excluded if they have cognitive impairment or serious comorbidities such as stroke or dementia.
Chen and Wu (2023) [18]	Taiwan	Single-blinded, randomized controlled trial	100 (50/50)/Participants must be >65 years, proficient in using mobile devices, and reading Chinese
Hadi Sulistyo et al. (2018) [19]	Indonesia	Two-group pre- and post-test quasi-experimental design	72 (35/37)/Participants must be aged between 18 and 65 years, diagnosed with diabetes mellitus, proficient in Indonesian, and independent. They should have no visual or hearing impairment and be reachable by telephone.
Ahmad Sharoni et al. (2018) [20]	Malaysia	Randomized controlled trial	71 (36/35)/Aged ≥60 years, diagnosed with diabetes, and able to communicate in Malay and be independent. They can have diabetic foot problems, but should not be blind, deaf and mute, or bedridden.
Moradi et al. (2019) [21]	Iran	Interventional quasi-experimental research design	160 (80/80)/Participants must be >30 years, diagnosed with type 2 diabetes for at least six months, and able to use mobile phones and send/receive text messages. They should have no history of diabetic foot ulcers and be willing to participate. Exclusions include severe psychiatric or cognitive problems, insulin therapy, major diabetic complications, or failure to attend educational sessions.
Tazangi et al. (2022) [22]	Iran	Randomized controlled clinical trial	70 (35/35)/Participants must be diagnosed with type 2 diabetes mellitus confirmed by endocrinologists, have a previous history or present clinical evidence of diabetic foot ulcers, and have the physical ability to participate in training. They should be able to speak, have no cognitive or behavioral disorders, and be willing to participate.
Abdelhamid, et al. (2018) [23]	Egypt	Quasi-experimental study design	70 (35/35)/Participants must be aged 18-65 years, reachable through telephone, and willing to participate. Exclusions include severe complications such as advanced diabetic retinopathy, acute vision or hearing impairment, disabilities, or joint diseases.
Wilbright et al. (2004) [24]	America	Non-randomized comparison	140 (20/120)/Louisiana State University Health Sciences Center Diabetes Foot Program, Baton Rouge, LA, and Lallie Kemp Medical Center, Independence, LA
Corbett (2003) [25]	America	Prospective, randomized, single-center, two-group design	40 (20/20)/Participants must be >18 years, able to participate physically and mentally, and read and understand English. They should have no ulcers, no history of lower limb amputation, and be receiving home care services.
Litzelman et al. (1993) [26]	America	Blinded, randomized, controlled trial	352 (191/205)/Participants must be >40 years, diagnosed with diabetes after age 30 based on National Diabetes Data Group criteria, and require medication for hyperglycemia control. They should intend to receive care at the general medicine practice for the next two years and be normal or overweight.
Monami et al. (2015) [27]	Italy	Randomized, open-label, single-center clinical trial	120 (60/60)/Participants must be >18 years, diagnosed with type 2 diabetes, and be at high risk for foot ulcers. Exclusions include peripheral vascular disease needing revascularization or cognitive impairment. They should have awareness of diabetes, but no specific foot care education.

Table 2 presents that among individuals identified as having a low risk for DFU, preventive interventions were predominantly implemented, encompassing multifaceted educational initiatives and routine follow-ups. Conceptual frameworks such as the Self-Efficacy Theory [17-19] and the Health Belief Model [22] were commonly employed to inform intervention strategies designed to influence awareness and behavioral engagement in foot care.

**Table 2:** Intervention details and outcome measures of the included studies FSCB: Foot Self-Care Behavior, SDSCA: Diabetes Self-Management Activity Summary Scale, FCCS: Foot Care Confidence Scale, Potentially Foot-Damaging Behavior, DFCK: Diabetic Foot Care Knowledge, DFCB: Diabetic Foot Care Behaviors, FCSE: Foot Care Self-Efficacy, KOFC: foot care outcome expectation, QoL: quality of life, FBS: fasting blood sugar, HbA1c: hemoglobin A1C, DQOL: Thomas Diabetes Quality of Life, BMI: body mass index

Reference	Intervention	Outcome measures	Outcome
[17]	Four-step self-efficacy program (performance achievement, vicarious experience, verbal persuasion, and physiological state), including 60- to 75-minute small-group education and practice session, foot care supplies, foot care kit, and three follow-ups through phone call over six months (weeks 2, 10, and 20)	FSCB, common skin conditions (dry skin, cracked skin, fungal infection, corns/calluses, redness, blisters, and minor lesions), toenail issues (hygiene, length, thickness, atrophy, trauma, inwardly grown toenails, and fungal infection); SDSCA, diabetes management (general diet, exercise, blood glucose monitoring, and medication); and FCCS	DCC (dry skin, cracked skin, and corn/callus) proportion decreased from 78% to 34.6% (p<0.001)
[18]	One-hour in-person session, weekly telephonic follow-up, feedback through mobile application, and technical support	Self-efficacy (Chinese version of Foot Care Self-Efficacy Scale), self-care behaviors (Diabetic Foot Self-Care Behavior Scale), and HbA1c monitoring	Significant improvements in self-efficacy, diabetes foot care behavior, and HbA1c (p<0.001)
[19]	One-hour lecture, academic video session, group discussion, practice session, and weekly telephonic counseling	DFCK and DFCB	Significant improvements in DFCK and DFCB scores (p<0.001)
[20]	Foot care kit (brochure, nail clippers, moisturizing lotion, and small towel), information on risk factors, hygiene, skin and nail care, appropriate footwear, injury prevention, and medical professional consultation	FSCB, FCSE, FCOE, knowledge of foot care (KOFC), and QoL	Improvements in self-care behavior, foot care self-efficacy, foot care outcome expectation, and knowledge of foot care (p<0.05); no significant improvements in QoL physical symptoms and psychosocial functioning
[21]	90 text messages per day for three months on DFU prevention behaviors	Preventive behaviors of DFU, monitoring of FBS^※9^, and HbA1c^※10^	Significant increase in DFU prevention behavior score (p<0.001); significant improvement in FBS score (p<0.001)
[22]	Five weeks of 45-minute educational sessions, peer group training (lectures, experience sharing, role-playing, group discussions, educational videos, pamphlets, and Q&A)	DQOL, self-care behavior	Significant improvements in self-care behavior, foot care knowledge, and self-efficacy (p<0.05)
[23]	Patients completed written questionnaires with the help of a researcher if needed, taking about 45 minutes; they had weekly follow-up appointments at the outpatient clinic and set goals and action plans over the telephone	Diabetic Foot Care Behaviors; duration of diabetes mellitus, random blood glucose levels, and diabetes treatment	All patients in the investigated group had satisfactory practices related to foot care after the intervention
[24]	Evaluation form sent prior to telemedicine consultations, assessing skin condition, wound condition, ulcer/wound grade, risk category, and treatment plan	Forefoot ulcer healing time, percentage of wounds healed, and healing time ratio	No significant differences in average forefoot ulcer healing time, percent of forefoot ulcers healed, and adjusted healing time ratio (p=0.828)
[25]	Home visit at six weeks, foot care education tailored to the patient's self-care behavior, repeated intervention at 12 weeks	Foot risk assessment, foot ulcer risk and foot care knowledge, foot care implementation status, and self-efficacy	Significant improvements in foot care knowledge, self-efficacy, and self-care behavior (p<0.05)
[26]	Foot care education, behavioral contract, and telephone and postcard reminders	Regular foot care routine and foot lesions (Seattle Wound Classification System)	Reduced incidence of serious foot lesions (p=0.05), improved self-foot-care behaviors
[27]	Two-hour program (30-minute one-to-one lesson, 90-minute interactive session)	Time spent on ulcer care, BMI, HbA1c, systolic pressure, and diastolic pressure	Trend toward reduction of HbA1c and BMI, no significant change in blood pressure levels

To deliver educational content and strengthen self-efficacy, interventions utilized both face-to-face methods and information and communication technology (ICT)-based approaches. To strengthen self-efficacy via verbal persuasion, a range of methods was utilized, including regular in-person visits by healthcare providers [20], telephone-based support [17-19], postcard reminders [26], and mobile health interventions, such as text messaging [21] and messaging applications [18]. Furthermore, peer group sessions integrating elements of vicarious learning and verbal encouragement [22] were conducted. Additional studies incorporated the implementation of short-term educational programs to evaluate the effectiveness of intensive, time-limited learning interventions [27], as well as real-time telemedicine consultations aimed at delivering effective care to patients with active ulcers who encounter financial or geographic barriers to accessing healthcare services [24].

Outcome Measures

The effectiveness of the interventions was assessed using outcome measures that captured changes in patients' behaviors and awareness, including adherence to foot care practices [17,19-23,25], knowledge of foot care, perceived self-efficacy [18,20], and overall quality of life [20,22]. Several studies assessed blood glucose levels and hemoglobin A1c status to evaluate the state of diabetes management [17-19,20]. Several studies evaluated behavioral modifications and the implementation of appropriate care routines by examining physical alterations in the lower extremities, such as the presence of calluses, corns, and nail hygiene [17]. Furthermore, investigations measured intervention efficacy by analyzing the amount of time healthcare professionals dedicated to DFU-related care [24]. Other studies focused on clinical outcomes in patients with active DFUs, evaluating the proportion of healed ulcers and the duration required for wound healing as indicators of treatment success [26,27].

Discussion

The purpose of this study was to elucidate non-pharmacological intervention measures implemented by healthcare professionals to prevent and manage DFU. This scoping review identified diverse intervention modalities, outcome measures, and delivery methods across 11 studies. The findings of this review emphasize that non-pharmacological interventions, particularly multidisciplinary educational programs involving nurses and other healthcare professionals, may be effective in preventing diabetic foot ulcers (DFUs) and preventing their recurrence. Consistent with previous research demonstrating the value of multidisciplinary team management [9], our review highlights that interactive, theory-based educational support delivered by nurses, podiatrists, and diabetes educators enhances patient self-efficacy, promotes foot care adherence, and improves clinical outcomes. The involvement of nurses is particularly crucial, as they provide continuous patient education, monitor foot health, facilitate behavior change, and coordinate care across healthcare settings. Future preventive strategies should prioritize the integration of nursing expertise within multidisciplinary teams to optimize DFU prevention and management.

To prevent the onset of DFU, non-face-to-face methods such as telephone consultations and information and communication technology (ICT) support were adopted. For patients at high risk of DFU onset or those already exhibiting symptoms, telemedicine was implemented to provide high-quality care regardless of geographical location. Key outcomes included measures of patients' quality of life following intervention, as well as assessments of foot care knowledge and behavior. Indirect indicators for preventing DFU onset included lower limb condition, presence of wounds, wound healing duration, and degree of metabolic control. Furthermore, the time spent on ulcer treatment and the duration of ulcer healing were also evaluated. However, the evaluation lacked consistency from the perspective of preventing DFU onset or recurrence, and a variety of outcome indicators were used.

Non-pharmacological preventive interventions for DFU primarily comprised educational strategies, with outcome measures focusing on behavioral modifications, knowledge acquisition, and quality of life enhancement. Previous studies have demonstrated that lifestyle-oriented interventions aimed at mitigating cardiovascular risk factors in individuals with diabetic foot pathology utilized outcome measures such as blood glucose levels and quality of life indices [28]. In alternative interventions aimed at preventing diabetes itself, blood glucose levels were predominantly used as the primary evaluative indicator [29]. In contrast to previous studies, the present investigation centered on preventing DFUs, utilizing behavioral changes and quality of life as outcome indicators, thereby aligning the observed outcomes with the specific characteristics of the implemented interventions. The variability in outcome measures, while presenting methodological challenges, reflects the multifaceted nature of DFU prevention, which necessitates evaluation through both biomarkers and behavioral indicators. Previous studies on decision-making for preventing foot ulcers have similarly highlighted this challenge [30]. Therefore, this underscores the importance of systematically evaluating outcomes in future preventive interventions targeting individuals at risk of developing diabetic foot ulcers.

The interventions primarily consisted of interactive educational support delivered through both face-to-face and ICT modalities. Approximately 80% of DFUs can be prevented through education; however, if education is ineffective, more intensive interventions or surgical treatment may be necessary [31]. In non-pharmacological preventive interventions for DFUs, the included studies employed interventive measures grounded in meso-level theories, such as self-efficacy and health belief models, to enhance confidence in self-care behaviors. Additionally, healthcare providers provided interactive support to patients that was continuous, bidirectional, and patient-centered rather than directive or unidirectional. In cases where the risk of DFUs is low, patients primarily engage in self-management at home. In recent years, the use of ICT, such as tablets and applications, has been promoted to enhance self-care support in the fields of chronic and home nursing care [21].

The importance of combining ICT with face-to-face educational support was suggested in diabetic foot ulcer (DFU) prevention interventions. This covers the fact that the educational interventions described in previous studies did not have a significant effect on ulceration and lower limb amputation rates [10]. The ability to see the patient's foot condition and self-care in real time has the advantage of being able to contact their healthcare provider as needed and receive ongoing assistance without having to visit a clinic. Previous studies on similar self-management approaches have also shown the utility of ICT [32].

Moving forward, emerging technologies such as artificial intelligence-based foot monitoring systems, wearable sensors for continuous gait and pressure assessment, and telemedicine platforms hold promise for enhancing DFU prevention by enabling earlier detection of risk factors and more personalized interventions [12,32]. However, research is needed to establish the optimal integration of these technologies with traditional face-to-face care, ensure cost-effectiveness and accessibility, and develop standardized protocols for their implementation across diverse healthcare settings.

Strengths and Limitations

This scoping review has the notable strength of comprehensively mapping non-pharmacological interventions for DFU prevention in patients with type 2 diabetes in diverse settings. By incorporating both international and Japanese databases, the review minimized language and publication bias, and highlighted a range of intervention modalities and outcome domains. However, this review has certain limitations. First, despite the comprehensive search strategy, the scope was restricted to published quantitative studies; relevant qualitative studies, grey literature, and unpublished reports were not included, possibly omitting valuable contextual insights. Second, the heterogeneity in study design, intervention components, and outcome measures limited the comparability of results across studies and precluded the conduct of a meta-analysis. Third, while a broad range of interventions was identified, the evaluation of preventive impact lacked consistency, as outcome indicators varied widely in terms of patient behavior, clinical outcomes, and healthcare provider involvement. Finally, due to the nature of scoping reviews, no assessment of risk of bias or study quality was conducted, which may affect the interpretability of findings.

## Conclusions

This study suggests potential benefits of non-pharmacological, educational interventions, delivered through both face-to-face and ICT-based modalities, in the prevention and post-onset management of DFUs. While the variability in outcome measures remains a limitation, the findings suggest potential benefits of incorporating both behavioral and quality of life indicators when evaluating such interventions. Educational strategies grounded in self-efficacy and health belief models, complemented by real-time interactive support, were central to promoting effective self-care practices among individuals at risk for DFUs. Importantly, multidisciplinary educational interventions involving nurses and other healthcare professionals appear to be particularly effective in preventing DFUs and preventing recurrence. To optimize preventive strategies, future research should aim to establish standardized outcome metrics and evidence-based guidelines for integrating face-to-face and ICT-supported multidisciplinary educational interventions.

## Appendices

Supplementary Table 1

Table 3 presents the checklist for the Preferred Reporting Items for Systematic Reviews and Meta-Analyses extension for Scoping Reviews.

**Table 3:** Preferred Reporting Items for Systematic Reviews and Meta-Analyses extension for Scoping Reviews checklist JBI: Joanna Briggs Institute, PRISMA-ScR: Preferred Reporting Items for Systematic Reviews and Meta-Analyses extension for Scoping Reviews *Where sources of evidence (see second footnote) are compiled from, such as bibliographic databases, social media platforms, and websites. †A more inclusive/heterogeneous term used to account for the different types of evidence or data sources (e.g., quantitative and/or qualitative research, expert opinion, and policy documents) that may be eligible in a scoping review as opposed to only studies. This is not to be confused with information sources (see first footnote). ‡The frameworks by Arksey and O'Malley (6) and Levac and colleagues (7) and the JBI guidance (4 and 5) refer to the process of data extraction in a scoping review as data charting. §The process of systematically examining research evidence to assess its validity, results, and relevance before using it to inform a decision. This term is used for items 12 and 19 instead of "risk of bias" (which is more applicable to systematic reviews of interventions) to include and acknowledge the various sources of evidence that may be used in a scoping review (e.g., quantitative and/or qualitative research, expert opinion, and policy document).

Section	Item	PRISMA-ScR checklist item	Reported on page #
Title			
Title	1	Identify the report as a scoping review	1
Abstract			
Structured summary	2	Provide a structured summary that includes (as applicable) background, objectives, eligibility criteria, sources of evidence, charting methods, results, and conclusions that relate to the review questions and objectives.	2
Introduction and background			
Rationale	1	Describe the rationale for the review in the context of what is already known and explain why the review questions/objectives lend themselves to a scoping review approach.	1-2
Objectives	2	Provide an explicit statement of the questions and objectives being addressed with reference to their key elements (e.g., population or participants, concepts, and context) or other relevant key elements used to conceptualize the review questions and/or objectives.	4-5
Methods			
Protocol and registration	5	Indicate whether a review protocol exists; state if and where it can be accessed (e.g., a Web address); and if available, provide registration information, including the registration number.	6-8
Eligibility criteria	6	Specify characteristics of the sources of evidence used as eligibility criteria (e.g., years considered, language, and publication status), and provide a rationale.	6
Information sources*	7	Describe all information sources in the search (e.g., databases with dates of coverage and contact with authors to identify additional sources), as well as the date the most recent search was executed.	6
Search	8	Present the full electronic search strategy for at least one database, including any limits used, such that it could be repeated.	6
Selection of sources of evidence†	9	State the process for selecting sources of evidence (i.e., screening and eligibility) included in the scoping review.	6
Data charting process‡	10	Describe the methods of charting data from the included sources of evidence (e.g., calibrated forms or forms that have been tested by the team before their use, and whether data charting was done independently or in duplicate) and any processes for obtaining and confirming data from investigators.	6
Data items	11	List and define all variables for which data were sought and any assumptions and simplifications made.	6
Critical appraisal of individual sources of evidence§	12	If done, provide a rationale for conducting a critical appraisal of included sources of evidence; describe the methods used and how this information was used in any data synthesis (if appropriate).	6
Synthesis of results	13	Describe the methods of handling and summarizing the data that were charted.	6
Results			
Selection of sources of evidence	14	Give numbers of sources of evidence screened, assessed for eligibility, and included in the review, with reasons for exclusions at each stage, ideally using a flow diagram.	9
Characteristics of sources of evidence	15	For each source of evidence, present characteristics for which data were charted and provide the citations.	9
Critical appraisal within sources of evidence	16	If done, present data on critical appraisal of included sources of evidence (see item 12).	9
Results of individual sources of evidence	17	For each included source of evidence, present the relevant data that were charted that relate to the review questions and objectives.	9
Synthesis of results	18	Summarize and/or present the charting results as they relate to the review questions and objectives.	9
Discussion			
Summary of evidence	19	Summarize the main results (including an overview of concepts, themes, and types of evidence available), link to the review questions and objectives, and consider the relevance to key groups.	10-13
Limitations	20	Discuss the limitations of the scoping review process.	10-13
Conclusions	21	Provide a general interpretation of the results with respect to the review questions and objectives, as well as potential implications and/or next steps.	13
Funding			
Funding	22	Describe sources of funding for the included sources of evidence, as well as sources of funding for the scoping review. Describe the role of the funders of the scoping review.	14

Supplementary Table 2

Table 4 presents the full search terms used in the study for each database.

**Table 4:** Search terms for each database

Database	Search terms
PubMed	#1 “Diabetes Mellitus”[mh] OR “Diabetes Mellitus, Type 2”[mh] OR “Diabetes Mellitus, Type II”[tiab] OR NIDDM[tiab] OR MODY[tiab]OR “Type 2 Diabetes Mellitus”[tiab] OR “Type 2 Diabetes” [tiab] OR “diabetic foot ulcer”[tiab] OR “Diabetes, Type 2”[tiab] #2 “Foot Ulcer”[mh] OR “Foot Ulcers”[tiab] OR “Ulcer, Foot”[tiab] OR “Ulcers, Foot”[tiab] OR “Plantar Ulcer”[tiab] OR “Plantar Ulcers”[tiab] OR “Ulcer, Plantar”[tiab] OR gangrene[tiab] OR “Diabetic Foot”[mh] OR “Foot, Diabetic”[tiab] OR “Diabetic Feet”[tiab] OR “Skin Ulcer”[mh] OR “Skin Ulcers”[tiab] OR “Ulcer, Skin”[tiab] OR “Ulcers, Skin”[tiab] OR “Critical Leg Ischemia”[tiab] OR “Chronic Limb-Threatening Ischemia”[tiab] #3 prevention[tiab] OR Implementation[tiab] OR dressing[tiab] intervention[tiab] OR Therap*[tiab] OR “Non pharmacologica”[tiab] Telemedicine[mh] Shoes[tiab]OR “Patient Education as Topic”[tiab] OR “Hyperbaric Oxygenation”[tiab] OR “Text Messaging”[tiab] OR Bandages[mh] OR Relaxation[tiab] OR “imagery intervention”[tiab] OR Honey[mh] #4 #1 AND #2 AND #3 #5 Animals[tiab] OR “Systematic Review”[tiab] OR “Guidelines as Topic”[mh] OR Review[Publication Type] OR Protocol[Title] #6 #4 NOT #5
Cochrane Central	#1 “Diabetes Mellitus”[mh] OR “Diabetes Mellitus, Type 2”[mh] OR “Diabetes Mellitus, Type II”[tiab] OR NIDDM[tiab] OR MODY[tiab]OR “Type 2 Diabetes Mellitus”[tiab] OR “Type 2 Diabetes” [tiab] OR “diabetic foot ulcer”[tiab] OR “Diabetes, Type 2”[tiab] #2 “Foot Ulcer”[mh] OR “Foot Ulcers”[tiab] OR “Ulcer, Foot”[tiab] OR “Ulcers, Foot”[tiab] OR “Plantar Ulcer”[tiab] OR “Plantar Ulcers”[tiab] OR “Ulcer, Plantar”[tiab] OR gangrene[tiab] OR “Diabetic Foot”[mh] OR “Foot, Diabetic”[tiab] OR “Diabetic Feet”[tiab] OR “Skin Ulcer”[mh] OR “Skin Ulcers”[tiab] OR “Ulcer, Skin”[tiab] OR “Ulcers, Skin”[tiab] OR “Critical Leg Ischemia”[tiab] OR “Chronic Limb-Threatening Ischemia”[tiab] #3 prevention[tiab] OR Implementation[tiab] OR dressing[tiab] intervention[tiab] OR Therap*[tiab] OR “Non pharmacologica”[tiab] Telemedicine[mh] Shoes[tiab]OR “Patient Education as Topic”[tiab] OR “Hyperbaric Oxygenation”[tiab] OR “Text Messaging”[tiab] OR Bandages[mh] OR Relaxation[tiab] OR “imagery intervention”[tiab] OR Honey[mh] #4 #1 AND #2 AND #3 #5 Animals[tiab] OR “Systematic Review”[tiab] OR “Guidelines as Topic”[mh] OR Review[Publication Type] OR Protocol[Title] #6 #4 NOT #5
Ichushi-Web	#1diabetes mellitus- type 2/TH OR type 2 diabetes mellitus/AL OR NIDDM/AL OR non-insulin-dependent diabetes mellitus/AL OR type II diabetes/AL OR non-insulin-dependent diabetes mellitus/AL #2 foot ulcer/TH OT foot infection/AL OR plantar ulcer/AL OR Foot Ulcer/AL OR ulcer of the foot/AL OR toe ulcer/AL OR foot ulcer/AL OR toe ulcer/AL OR diabetic foot symptoms/TH OR diabetic foot ulcer/aL OR Diabetic Foot Ulcer/AL OR diabetic foot ulcer/AL OR diabetic foot lesion/AL OR diabetic toe ulcer/AL OR diabetic gangrene/TH OR diabetes gangrene/AL OR lower limb ulcer/AL OR foot ulcer/TH OR plantar ulcer/AL OR ulcer/TH #3 relaxation therapy/TH OR acupressure/TH OR soft tissue treatment/TH OR foot bath/TH OR dressing with biological material/TH OR dressing/TH OR hyperbaric oxygenation/TH OR diabetes education/TH OR footwear/TH OR stocks/stockings/TH OR arch support/TH OR team treatment/TH OR physician-nurse relationship/TH OR nursing technique/TH OR patient education/TH OR health education/TH OR preventive medicine/TH OR hydrocolloid dressing materials/TH OR thermography/TH OR carbon dioxide/TH OR remote medicine/TH #4 #1 AND #2 AND #3 #5 fact-finding investigation/TH OR literature study/TH #6 #4 NOT #5
